# Peri-Carotid Adipose Tissue and Atherosclerosis at Carotid Bifurcation

**DOI:** 10.3390/jcdd11020058

**Published:** 2024-02-11

**Authors:** Joana Ferreira, Adhemar Longatto-Filho, Ana Dionísio, Margarida Correia-Neves, Pedro Cunha, Armando Mansilha

**Affiliations:** 1 Vascular Surgery Department, Physiology and Surgery, University Hospital Centre of São João, 4200-319 Porto, Portugal; 2 Academic Centre of Hospital Senhora da Oliveira, 4835-044 Guimarães, Portugal; 3 Life and Health Science Research Institute (ICVS), School of Medicine, University of Minho, 4710-057 Braga, Portugalpedrocunha@med.uminho.pt (P.C.); 4 ICVS/3B’s-PT Government Associate Laboratory, 4710-057 Braga, Portugal; 5 Department of Pathology (LIM-14), Faculty of Medicine of the University of São Paulo, São Paulo 01246 903, Brazil; 6 Molecular Oncology Research Centre, Barretos Cancer Hospital, São Paulo 14784-400, Brazil; 7 Porto Vascular Conference Scientific Advising, 4050-430 Porto, Portugal; 8 Medicine Department of Hospital Senhora da Oliveira, 4835-044 Guimarães, Portugal; 9 Centre for the Research and Treatment of Arterial Hypertension and Cardiovascular Risk, Internal Medicine, 4835-044 Guimarães, Portugal; 10 Faculty of Medicine of the University of Porto, 4200-319 Porto, Portugal

**Keywords:** atherosclerosis, carotid stenosis, perivascular adipose tissue, “acute culprit”, carotid stenosis, extra-media thickness

## Abstract

Vulnerable carotid plaques are responsible for 20% of the ischemic strokes. The identification of these asymptomatic carotid plaques that will become symptomatic is essential but remains unclear. Our main goal was to investigate whether the amount of the peri-carotid adipose tissue, estimated by the extra-media thickness (EMT), is associated with the atherosclerotic characteristics at the carotid bifurcation in patients with PAD. An observational, prospective, single-center, longitudinal study was conducted. Overall, 177 patients were subjected to carotid Doppler ultrasound at the study admission. The following data were collected: EMT, intima-media thickness (IMT), the presence of carotid plaques, the area of the highest plaque, the presence of “acute culprit” carotid stenosis, and the grade of internal carotid stenosis. “Acute culprit” carotid stenosis was defined as a significant atherosclerotic plaque that leads to a neurologic event within 15 days. From each carotid bifurcation, a right and a left EMT were determined. We analyzed both the mean EMTs (calculated as the mean between the right and the left EMT) and the EMT ipsilateral to the carotid bifurcation. The presence of carotid plaques was associated with a higher mean EMT [Median = 1.14; IQR = 0.66 versus Median = 0.97; IQR = 0.40; *p* = 0.001]. A positive correlation was found between the mean EMT and IMT (right: ρ = 0.20; *p* = 0.010; left: ρ = 0.21; *p* = 0.007) and between the mean EMT and the area of the largest carotid plaque (right: ρ = 0.17; *p* = 0.036; left: ρ = 0.22; *p* = 0.004). Left carotid stenosis ≥ 70% was associated with higher ipsilateral EMT [Median = 1.56; IQR = 0.70 versus Median = 0.94; IQR = 0.42; *p* = 0.009]. Patients with “acute culprit” carotid stenosis had a higher ipsilateral EMT [left ipsilateral EMT: Median = 1.46; IQR = 0.63; “non-acute”: Median = 0.94; IQR = 0.43; *p* = 0.009; right ipsilateral EMT: Median = 2.25; IQR = 0.62; “non-acute”: Median = 1.00; IQR = 0.51; *p* = 0.015]. This difference was not found in the contra-lateral EMT. Six months after the neurologic event, EMT ipsilateral to an “acute culprit” carotid stenosis decreased (*p* = 0.036). The amount of peri-carotid adipose tissue, estimated with EMT, was associated with atherosclerosis at the carotid arteries. The mean EMT was associated with the features of chronic atherosclerosis lesions: the presence of carotid plaques, IMT, and the area of the highest plaque. Ipsilateral EMT was linked with “acute culprit” atherosclerotic plaque.

## 1. Introduction

Stroke is the second cause of death in Europe among cardiovascular diseases, with 1.4 million people experiencing a stroke every year [1]. Acute ischemic stroke accounts for roughly 80% of all strokes, and 20% of which originate in vulnerable carotid plaques [2]. The identification of these asymptomatic carotid plaques that will become vulnerable and symptomatic remains unclear, despite its value in receiving an earlier intervention. Several biomarkers have been explored to identify asymptomatic carotid patients who could benefit from prophylactic carotid intervention [3]. Recognizing the importance of predicting which plaques will become symptomatic, the European Society for Vascular Surgery Guidelines have emphasized “the need for a validated algorithm for identifying high risk for stroke on best medical therapy asymptomatic patients in whom to target carotid endarterectomy and carotid artery stenting” [1].

Studies conducted in cardiology reported that changes in coronary perivascular adipose tissue (PVAT) are associated with vulnerable coronary plaques and can be identified using computed tomography [4,5,6].

PVAT is the adipose tissue that supports the vessels and produces several molecules that affect the function and structure of the vessels [7,8]. The relationship between the PVAT and the vessels is complex and bidirectional [9]. The PVAT exerts anti-inflammatory and vasodilatory functions, which have a protective effect [8,10]. However, under pathological conditions, the PVAT can become dysfunctional, leading to an increase in vasoconstriction agents and to a proinflammatory phenotype, and may ultimately be involved in the initiation and progression of atherosclerosis [8,10]. 

The amount of PVAT that surrounds the common carotid artery can be easily measured in clinical practice using ultrasound, with carotid artery extra-media thickness (EMT) [11]. The main determinants of EMT are arterial adventitia and PVAT [7,11].

The primary aim of this study was to analyze whether the amount of the peri-carotid adipose tissue, estimated through EMT, is associated with the atherosclerotic characteristics at the carotid bifurcation. 

## 2. Materials and Methods

### 2.1. Study Type and Inclusion/Exclusion Criteria

An observational, prospective, unicenter study was conducted from January 2018 to December 2022. Patient recruitment rate was reduced during 2020 due to the COVID-19 pandemic. We included 106 consecutive patients who were attending the Vascular Surgery consultations with the first author (JF) for varicose veins, peripheral arterial disease, and carotid artery plaque. Patients with symptomatic carotid stenosis included in the study were admitted to the stroke unit and assessed by both the neurologist and the first author of this paper. These patients were followed-up for six months by the first author (JF). Bedridden individuals, subjects who refused to participate in the protocol, or patients with any disease that could potentially change the body composition or the pro-inflammatory state (e.g., recent diet change, active malignancy, auto-immune disease, active infection, chronic renal failure with glomerular filtration rate of <30 mL/min/1.73 m^2^ and heart failure in the past three months) were excluded.

### 2.2. Ethical Considerations

Ethics approval was obtained from the Ethics Committee of the local hospital, Senhora da Oliveira in Guimarães (Ref:75/2017). All participants signed the informed consent. 

### 2.3. Demographic and Clinical Characteristics

Patient’s age, gender, and medication were registered. The definitions of arterial hypertension, diabetes, dyslipidemia, and smoking habits were considered according to other publications [12]. Obesity was defined as waist circumference (WC) > 102 cm in men and >88 cm in women [13]. “Acute culprit” carotid stenosis was defined as the presence of an extracranial atherosclerotic plaque that was higher than 50%, leading to symptoms of amaurosis fugax, transient ischemic attacks, or ischemic stroke ipsilateral to the lesion and identified after and within the first 15 days the neurologic event [14,15,16]. 

### 2.4. Lipid Profile and HbA1c

To determine the levels of hemoglobin A1c (HbA1c), total cholesterol, high-density lipoprotein (HDL), low-density lipoprotein (LDL), and triglycerides, a phlebotomy was performed in the morning, after a 10–12 h fast. Blood samples were collected into appropriate Vacutainers, centrifuged for 5 min at 4000 cycle/minute rate, and the serum was separated. The tests were performed by routine procedures in the department of clinical chemistry.

### 2.5. Evaluation of the Carotid Bifurcation with Doppler Ultrasound

The carotids were characterized using a SIEMENS (Siemens Medical Solutions, Inc., Mountain View, CA, USA) ACUSON X300 ultrasound, with a 5–10 Hz linear scan head. A two-dimensional gray scale was used to measure the intima-media thickness (IMT), the EMT, and the presence and morphology of the atherosclerotic plaque [7].

To determine the IMT, the probe surface was placed parallel to the common carotid artery, in a longitudinal position, and is the standard method to assess the carotid arteries [17]. Two bright lines were identified, i.e., the interface between blood and the intima and the layer between intima and media [17], with the distance between these two lines being defined as the IMT [17]. The IMT was measured according to the Mannheim Consensus guidelines on a 10 mm length segment starting 5 mm proximal to the carotid bulb. Three measurements were taken for each common carotid artery, and their mean value was the IMT [18].

To determine the EMT, the carotid artery and jugular vein were both scanned in a longitudinal position with the probe parallel to the vessels [7]. The distance between the carotid media-adventitia interface and the jugular lumen was determined along the 7 mm segment, starting 3 mm proximal to the bulb [7]. Five values were recorded at the end-diastole, from five consecutive beats. The mean of these values indicated the EMT [18]. For each carotid bifurcation, right and left EMTs were determined, to evaluate the mean EMT (calculated as the mean between the right and the left EMT), and the EMT ipsilateral to the carotid bifurcation. To determine if the amount of peri-carotid adipose tissue correlated with the ipsilateral carotid atherosclerotic characteristics, we compared the EMT on the left and right sides to the ipsilateral and contralateral characteristics of atherosclerosis at the carotid bifurcation. 

The number of atherosclerotic plaques at the carotid bifurcation was recorded. The morphology and the area of the largest plaque were described. The plaque surface was classified as smooth, irregular, or ulcerated. A depression of the plaque surface by more than 2 mm indicates ulceration [18]. The plaque area was calculated based on the length and height of the plaque [18]. The plaque echogenicity was classified according to the Gray-Weale’s classification modified by Geroulakos [19].

Color and pulse Doppler ultrasonography was applied to determine the grade of carotid artery stenosis, according to the Guidelines of the European Society of Vascular Surgery [1,18].

### 2.6. Power Consideration

To determine the differences in the amount of the peri-carotid adipose tissue estimated through EMT between patients with carotid plaques and those without carotid plaques (a marker of atherosclerosis at the carotid bifurcation), a minimum sample of 106 patients is necessary (with a significant level of 5%, a power of 80%, and an effect size of 0.5).

### 2.7. Statistical Analysis

Continuous variables were expressed as the mean ± standard deviation, while percentages were presented for categorical variables. The Shapiro–Wilk test was used to assess all continuous variables for normality. Between-group differences in continuous variables were assessed with the Mann–Whitney U test, and the effect size r was determined by calculating the ratio between test statistics Z and the square root of the number of pairs n. Associations between parameters were assessed using the non-parametric Spearman correlation analysis since variables were not normally distributed. The correlations were corrected for the Bonferroni effect. Wilcoxon Signed Rank test was used to study the evolution of EMT, comparing 2 time points (t = 0 and t = 6 months). A *p*-value of less than 0.05 was considered statistically significant. Statistical analysis was performed using the SPSS Statistics software, version 20.0 (IBM SPSS Statistics, IBM Corporation Chicago, Chicago, IL, USA).

## 3. Results

### 3.1. General Description of the Population

We included 177 subjects (mean age: 67.16 ± 9.87 years; 79.66% males). This population had a higher prevalence of cardiovascular risk factors, with hypertension being the most prevalent followed by smoking habits and dyslipidemia. Notably, the intake of statins and antiplatelet therapy was substantial (Table S1). The atherosclerotic characteristics at the right and left carotid bifurcations are presented in Supplementary Materials (Table S2).

### 3.2. Peri-Carotid Adipose Tissue and Cardiovascular Risk Factors

The majority of the included patients had at least three cardiovascular risk factors (34.6% had three and 24.0% had four risk factors).

Patients with hypertension and patients with obesity had a higher mean EMT, whereas patients on fibrates had a lower mean EMT (Table 1). Age and the number of cardiovascular risk factors showed a weak positive correlation with the mean EMT (rs = 0.21; *p* = 0.006 and rs = 0.24; *p* = 0.002). 

### 3.3. Peri-Carotid Adipose Tissue and Chronic Atherosclerosis at the Carotid Bifurcation

#### 3.3.1. Presence of Carotid Plaques

Mean EMT: the presence of carotid plaques was associated with a statistically significant increase in mean EMT [present: Median = 1.14, IQR = 0.66; versus non-present: Median = 0.97, IQR = 0.40; *p* = 0.001, r = 0.26]. 

Ipsilateral EMT: Patients with plaques at the right carotid arteries were associated with a significantly higher ipsilateral EMT [Median = 1.02; IQR = 0.58; versus Median = 0.96; IQR = 0.48; *p* < 0.001]. This association was not found on the left side. The left EMT was also significantly higher in patients with right carotid plaques [Median = 0.93; IQR = 1.60; versus Median = 0.80; IQR = 1.20; *p* = 0.010]. This association persisted even after a correction for the Bonferroni effect.

#### 3.3.2. Area of Highest Carotid Plaque

Mean EMT: A positive correlation was found between the mean EMT and the area of the highest carotid plaque, at both right and left sides (ρ = 017; *p* = 0.036; ρ = 0.22; *p* = 0.004, respectively). The association persisted at the left side even after a correction for the Bonferroni effect.

Ipsilateral EMT: A positive correlation was found between the left EMT and the ipsilateral area of the carotid plaque (ρ = 0.22; *p* = 0.004). This association was not found on the right side. There was no correlation between the right EMT and the area of the contralateral carotid plaque.

#### 3.3.3. IMT

Mean EMT: a positive correlation was found between the mean EMT and IMT (right and left) (right: rs = 0.20; *p* = 0.010; left: rs = 0.21; *p* = 0.007). 

Ipsilateral EMT: a positive correlation was found between the ipsilateral EMT and the ipsilateral IMT (right: ρ = 0.19; *p* = 0.017; left: ρ = 0.19; *p* = 0.012). 

#### 3.3.4. Carotid Stenosis of ≥70%

Mean EMT: The presence of a left carotid stenosis of ≥70% was associated with a statistically significant increase in the mean EMT [present: Median = 1.55, IQR = 0.68 versus non-present: Median = 1.00, IQR = 0.46; *p* = 0.019, r = 0.61]. The association disappeared after a correction for the Bonferroni effect.

Ipsilateral EMT: patients with a left carotid stenosis of ≥70% were associated with a statistically significant and higher ipsilateral EMT (Table 2).

#### 3.3.5. Plaque Echogenicity

There were no differences in EMT (mean or ipsilateral) with respect to the echogenicity of carotid plaques (Tables S3 and S4).

### 3.4. The Particular Case of “Acute Culprit” Carotid Stenosis

We found that patients with “acute culprit” carotid stenosis (*n* = 11) had a significantly higher mean EMT and ipsilateral EMT, when compared to those with non-acute carotid stenosis patients. This finding revealed a large effect size and was valid for both left and right carotid stenoses (Table 3). This association was not found in the contra-lateral carotid bifurcation. However, after correcting for the Bonferroni effect, the association was just evident for the ipsilateral EMT and not for the mean EMT.

No significant differences were identified in the cardiovascular risk factors between patients with acute and non-acute culprit carotid stenoses.

**Figure 1:**
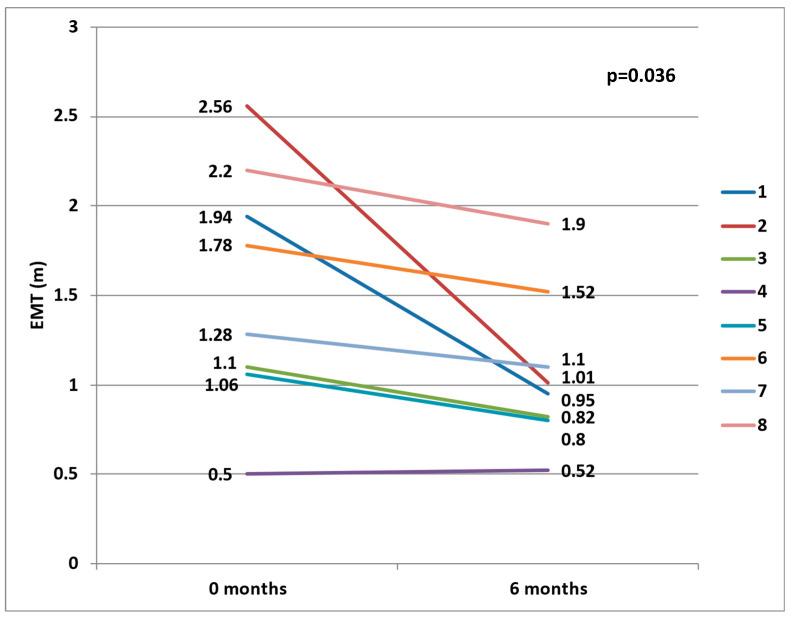
The evolution of ipsilateral EMT in “acute culprit” carotid stenosis, at the six-month follow-up.

Furthermore, we evaluated the ipsilateral peri-carotid adipose tissue six months after the neurologic event, by measuring a follow-up EMT, in 8 of 11 patients with “acute culprit” carotid stenosis, and we found a significant decrease in the ipsilateral EMT, both in right and left stenoses (Figure 1).

## 4. Discussion

To the best of our knowledge, our group is the first group trying to elucidate the role of ipsilateral peri-carotid adipose tissue versus the total amount of such tissue in atherosclerosis at the carotid bifurcation. Our research has revealed a correlation between the total amount of peri-carotid adipose tissue, estimated with the mean EMT, and some feature of chronic atherosclerosis lesions in the carotid arteries: the presence of carotid plaque, IMT, and the area of the highest plaque. In patients with “acute culprit” carotid stenosis, a strong link was found with the ipsilateral peri-carotid adipose tissue, potentially serving as a marker of acute events.

### 4.1. Peri-Carotid Adipose Tissue and Cardiovascular Risk Factors

We found that patients with obesity or with hypertension had a higher amount of peri-carotid adipose tissue, estimated by the mean EMT. Various authors have demonstrated that EMT is associated with the major parameters of obesity, including abdominal obesity [7,8,20]. Pacifico et al. have highlighted the expansion of peri-carotid adipose tissue in obesity and emphasized the role of visceral obesity in vascular disease at distant sites [8]. Obesity increases the amount of PVAT and causes functional changes, leading to vascular remodeling [7,21].

It is well established in the literature that there is a recognized association between a higher EMT and hypertension, independent of obesity [8,20,22]. EMT is a measure of carotid PVAT, which produces molecules that modulate the vascular tone [23].

We also found that a higher mean EMT was correlated with aging and with a higher number of cardiovascular risk factors, in accordance with the previous reports [7,20,22,24]. However, contrary to all other studies, we did not find any association between EMT and diabetes or between EMT and lipid profile [7,20]. These differences may, in part, be explained by the fact that the studies analyzed different populations [7,20]. We studied patients from the Vascular Surgery department, while Haberka et al. studied patients from the Cardiology department and Skilton et al. included a control group [7,20]. Another possible explanation could be the variance in the number of included patients (177 in our study compared to 400 in Haberka’s study) and the absence of risk factor control analysis in any of these papers. 

The physiopathological explanation for the possible association between diabetes and an increase in EMT (a measure of PVAT) is that an increase in PVAT causes microvascular dysfunction in muscle and consequently insulin resistance [25].

Remarkably, our study revealed that patients on fibrates had a lower mean EMT, which has not been previously described. This finding aligns with the documented evidence, indicating that fibrates inhibit adipocyte hypertrophy, offering a potential explanation for our results [26]. Further studies are needed to support this novel finding that suggests a potential protective effect of fibrates on diseases with abnormally increased EMT. Additionally, the literature supports that statins can limit the adventitial neovascularization and the EMT progression [24,27]. However, In our study, we observed no effect of statins on the mean EMT.

### 4.2. Peri-Carotid Adipose Tissue and Chronic Atherosclerosis at the Carotid Bifurcation

We found that the total amount of peri-carotid adipose tissue, estimated with the mean EMT, was associated with the presence of carotid plaques and with IMT. Haberka et al. also observed a significant, albeit weak association, between EMT and IMT, suggesting that both indexes may share underlying factors, with EMT being more related to the cardiometabolic risk [7].

Patients with left carotid stenosis of ≥70% had a statistically significant and higher ipsilateral EMT, with a large effect size. Our study is unique in separately analyzing stenosis at the right and left bifurcations [7,11]. 

We identified only one study that also analyzed the association between EMT and carotid stenosis, which included a population scheduled for elective coronary angiography [11]. This study demonstrated that the mean EMT was a strong and independent predictor of the severity of internal carotid stenosis, surpassing the predictive power of other cardiovascular risk factors [11]. The authors suggested that the mean EMT may be a systemic marker of atherosclerosis [11]. While the literature analyzing the peri-carotid adipose tissue is still scarce, the evidence on the peri-coronary adipose tissue is more elucidative. Several studies have demonstrated that the volume of peri-coronary epicardial adipose tissue was significantly associated with the extent and severity of coronary atherosclerosis [28,29].

No previous study has analyzed the relationship between the ipsilateral/contralateral EMT and the atherosclerotic characteristics at the carotid bifurcation, limiting our ability to discuss our results. It seems that the peri-carotid adipose tissue, estimated with EMT, may not have a direct local ipsilateral effect in a chronic atherosclerotic lesion at the carotid bifurcation. This aligns with Haberka et al.’s argument that EMT, which is measured in the distal segment of the common carotid artery, may be a systemic marker of atherosclerotic and not have an ipsilateral action in the carotid bulb/proximal internal carotid artery [11].

### 4.3. The Particular Case of “Acute Culprit” Carotid Stenosis

We observed a higher ipsilateral EMT for an “acute culprit” carotid stenosis at the left and right carotid bifurcations (when compared to patients with non-acute carotid stenosis or to the contra-lateral side). Six months after the neurologic event, the proportion of EMT ipsilateral to an “acute culprit” carotid stenosis decreased, which suggests a resolution mechanism following the acute phase. 

To our knowledge, this is the first time that such results have been described with Doppler ultrasound. Baradan et al. demonstrated an increase in the density of the peri-carotid adipose tissue surrounding the stenotic internal carotid arteries in patients with a history of ipsilateral stroke or transitory ischemic attack, using CT scans [30]. A higher fat density on a CT scan was associated with the histopathological markers of inflammation [10,30]. The authors suggested that the inflammation associated with “culprit” carotid plaques extends beyond the vessel lumen [30]. 

As previously mentioned, a higher number of publications are found on coronary disease, with Balcer et al. reporting a higher volume of peri-coronary adipose tissue surrounding a culprit coronary stenosis [31]. Inflammatory changes in the fat surrounding coronary arteries have been associated with coronary artery disease and with high-risk, rupture-prone “culprit” plaques [30]. Culprit lesions in patients with acute coronary syndrome exhibit an increase in inflammatory activity [32]. Moreover, peri-vascular adipose tissue depots expand through differentiation or in response to the infiltration of inflammatory cells and have a complex bidirectional relationship with the vascular wall [11]. Thus, we hypothesize that our observation of an augmented EMT ipsilateral to an “acute culprit” carotid stenosis could reflect an increased inflammation in peri-carotid adipose tissue associated with “acute culprit” carotid plaques. However, the question remains unanswered whether the inflammation in PVAT is a cause or a consequence of atherosclerosis [33].

### 4.4. Peri-Carotid Adipose Tissue as a Marker of Plaque Instability in Clinical Practice

The interest in identifying features related to carotid plaque instability is growing, along with the investigation of several biomarkers [34]. Although the markers of systemic inflammation, such as C-reactive protein, have been associated with an increased risk of plaque instability, they cannot specifically identify unstable or higher-risk vascular beds [30]. Peri-carotid adipose tissue may serve as a local biomarker of higher stroke risk, similar to the adipose tissue surrounding the coronary arteries, which has been associated with an increased risk of fatal heart attacks [9]. Furthermore, cross-sectional imaging techniques can assess peri-carotid tissue inflammation and identify the possible “culprit” carotid stenosis [9,30]. However, these imaging methods are time-consuming, expensive, and less readily available in clinical practice. Ultrasound, in turn, is an attractive, non-invasive method that can be easily performed at the patient’s bedside. 

Our results hypothesized that EMT may increase before acute carotid events and may be able to help in predicting them, aiding in clinical risk stratification and monitoring the therapeutic effect of anti-atherosclerotic medication. As the EMT ipsilateral to an “acute culprit” carotid stenosis decreases with time, reflecting a decrease in the inflammatory activity, this may point to the optimal timing for carotid endarterectomy. However, these are merely hypotheses that require validation through animal studies or larger-scale investigations.

### 4.5. Strengths and Limitations

This paper has several strengths. It is a prospective, longitudinal study that includes 177 patients. It is one of the few investigations analyzing the association between peri-carotid adipose tissue and atherosclerosis at the carotid bifurcation. It conducts a distinct analysis between the total and the ipsilateral PVAT. To the best of our knowledge, it is the first study demonstrating a significant association between an increased amount of ipsilateral peri-carotid adipose tissue, estimated with EMT, and “acute culprit” carotid stenosis. This finding is clinically significant as the measurement of peri-carotid adipose tissue using Doppler ultrasound is easily obtainable in daily practice and can eventually have implications on personalized treatment approaches, such as determining the need for carotid endarterectomy in borderline cases. However, the clinical applicability of EMT should be approached with caution and compared with robust studies. This paper also reports a previously undocumented observation that patients on fibrates had a lower EMT, suggesting a potential protective effect of fibrates on carotid diseases, although further research is needed to confirm this finding.

However, this study has several limitations. The sample under study does not fully represent the entire population followed-up at our institution but consists of consecutive patients followed-up by the main author. We defined the inclusion criteria to encompass patients who did not exhibit a pro-inflammatory state or any recent changes in their body composition. This approach aims to mitigate the potential biases in the results, considering the established connection between inflammation and the prevalence and instability of atherosclerotic plaque. This fact prevents our population from being similar to a real-life counterpart. Only 11 out of the total 177 patients had an “acute culprit” carotid stenosis. The use of Doppler ultrasound, a two-dimensional imaging method, can also compromise the evaluation of three-dimensional structures, such as carotid arteries. It is likely that the number of plaques could not be accurately quantified with this method as some of them appeared continuous. A larger sample size from multiple centers could provide more robust and generalizable results. Another limitation is the relatively short follow-up period of six months for “acute culprit” carotid stenosis. Furthermore, this study is unable to establish a causal relationship between peri-carotid adipose tissue and atherosclerosis at the carotid bifurcation.

## 5. Conclusions

Peri-carotid adipose tissue estimated with EMT is associated with the chronic marker of atherosclerosis at the carotid bifurcation and is also linked with “acute culprit” carotid stenosis.

## Figures and Tables

**Table 1:** Cardiovascular risk factors, medication, and the mean EMT.

	Present		Non-Present		U	*p*-Value	r
	EMT (mm)		EMT (mm)				
	Median	IQR	Median	IQR			
Cardiovascular risk factors							
Hypertension	1.07	0.49	0.90	0.37	2165	0.002	0.24
Smoker/ex-smoker	1.00	0.44	1.03	0.51	3068	0.602	0.04
Dyslipidemia	1.04	0.48	0.99	0.52	3102	0.337	0.07
Diabetes	1.00	0.45	1.02	0.47	3206	0.530	0.05
Obesity *	1.06	0.51	0.91	0.45	2336	0.006	0.21
Medication							
Fibrate	0.86	0.20	1.04	0.53	641	0.028	−0.17
Statins	1.04	0.52	0.97	0.67	1994	0.395	−0.06
Antiplatelet	1.04	0.47	0.99	0.74	2797	0.950	−0.04
ACEi/ARBs	1.04	0.64	1.00	0.50	2845	0.557	−0.04

EMT: extra-media thickness; ACEi/ARBs: angiotensin-converting enzyme inhibitors/angiotensin receptors blockers; and * Obesity defined as WC > 102 cm in men and WC > 88 cm in women.

**Table 2:** EMT and carotid stenosis of ≥70%.

Carotid Stenosis of ≥70%	Present		Non-Present		U	*p*-Value	r
	Median	IQR	Median	IQR			
Left carotid stenosis							
ipsilateral EMT	1.56	0.70	0.94	0.42	234	0.009	0.68
Right carotid stenosis							
ipsilateral EMT	1.10	1.12	1.00	0.53	734	0.261	0.33

EMT: extra-media thickness.

**Table 3:** EMT and “acute culprit” carotid stenosis at the left and right sides.

	Acute		Non-Acute		U	*p*-Value	r
	Median	IQR	Median	IQR			
Left carotid stenosis							
mean EMT	1.56	0.57	1.00	0.46	169	0.025	0.91
ipsilateral EMT	1.46	0.63	0.94	0.43	127	0.009	0.95
contralateral EMT	1.55	0.72	1.00	0.51	202	0.211	0.51
Right carotid stenosis							
mean EMT	1.82	0.24	1.00	0.47	23	0.021	0.94
ipsilateral EMT	2.25	0.62	1.00	0.51	18	0.015	0.96
contralateral EMT	1.54	1.40	0.96	0.38	111	0.444	0.37

EMT: extra-media thickness.

## Data Availability

The data that support the findings of this study are available from the corresponding author (J.F.).

## Supplementary Materials

The following supporting information can be downloaded at: https://www.mdpi.com/article/10.3390/jcdd11020058/s1. Table S1: Cardiovascular risk factors and comorbidities of the studied population; Table S2: The atherosclerotic characteristics at the right and left carotid bifurcations: the grade of carotid stenosis, the type of carotid atherosclerotic plaque, and the type of plaque surface; Table S3: The mean EMT and the echogenicity of carotid plaques at the right and left carotid bifurcations; and Table S4: The ipsilateral EMT at the right and left sides and the echogenicity of the right and left carotid plaques.

## References

[1] Naylor R., Rantner B., Ancetti S., de Borst G.J., De Carlo M., Halliday A., Kakkos S.K., Markus H.S., McCabe D.J.H., Sillesen H. (2023). Editor’s Choice—European Society for Vascular Surgery (ESVS) 2023 Clinical Practice Guidelines on the Management of Atherosclerotic Carotid and Vertebral Artery Disease. Eur. J. Vasc. Endovasc. Surg..

[2] Zhang Y., Cao J., Zhou J., Zhang C., Li Q., Chen S., Feinstein S., Grayburn P.A., Huang P. (2021). Plaque Elasticity and Intraplaque Neovascularisation on Carotid Artery Ultrasound: A Comparative Histological Study. Eur. J. Vasc. Endovasc. Surg..

[3] Paraskevas K.I., Veith F.J., Spence J.D. (2018). How to identify which patients with asymptomatic carotid stenosis could benefit from endarterectomy or stenting. Stroke Vasc. Neurol..

[4] Zhang S., Yu X., Gu H., Kang B., Guo N., Wang X. (2022). Identification of high-risk carotid plaque by using carotid perivascular fat density on computed tomography angiography. Eur. J. Radiol..

[5] Sugiyama T., Kanaji Y., Hoshino M., Yamaguchi M., Hada M., Ohya H., Sumino Y., Hirano H., Kanno Y., Horie T. (2020). Determinants of Pericoronary Adipose Tissue Attenuation on Computed Tomography Angiography in Coronary Artery Disease. J. Am. Heart Assoc..

[6] Honold S., Wildauer M., Beyer C., Feuchtner G., Senoner T., Jaschke W., Gizewski E., Bauer A., Friedrich G., Stühlinger M. (2021). Reciprocal communication of pericoronary adipose tissue and coronary atherogenesis. Eur. J. Radiol..

[7] Haberka M., Gąsior Z. (2015). Carotid extra-media thickness in obesity and metabolic syndrome: A novel index of perivascular adipose tissue: Extra-media thickness in obesity and metabolic syndrome. Atherosclerosis.

[8] Pacifico L., Perla F.M., Tromba L., Carbotta G., Lavorato M., Pierimarchi P., Chiesa C. (2020). Carotid Extra-Media Thickness in Children: Relationships with Cardiometabolic Risk Factors and Endothelial Function. Front. Endocrinol..

[9] Zhang D.-H., Jin J.-L., Zhu C.-F., Chen Q.-Y., He X.-W. (2021). Association between carotid artery perivascular fat density and cerebral small vessel disease. Aging.

[10] Liu Y., Xu L., Gu Y., Zhang Y., Miao C. (2021). Impact of H-Type Hypertension on Pericarotid Adipose Tissue and Plaque Characteristics Based on Computed Tomography (CT) Angiography: A Propensity Score Matching Study. J. Med. Sci. Monit..

[11] Haberka M., Skilton M., Biedroń M., Szóstak-Janiak K., Partyka M., Matla M., Gąsior Z. (2019). Obesity, visceral adiposity and carotid atherosclerosis. J. Diabetes Its Complicat..

[12] Ferreira J., Carneiro A., Vila I., Silva C., Cunha C., Longatto-Filho A., Mesquita A., Cotter J., Mansilha A., Correia-Neves M. (2023). Inflammation and Loss of Skeletal Muscle Mass in Chronic Limb Threatening Ischemia. Ann. Vasc. Surg..

[13] Nishida C., Ko G.T., Kumanyika S. (2010). Body fat distribution and noncommunicable diseases in populations: Overview of the 2008 WHO Expert Consultation on Waist Circumference and Waist–Hip Ratio. Eur. J. Clin. Nutr..

[14] Ohara T., Toyoda K., Otsubo R., Nagatsuka K., Kubota Y., Yasaka M., Naritomi H., Minematsu K. (2008). Eccentric stenosis of the carotid artery associated with ipsilateral cerebrovascular events. Am. J. Neuroradiol..

[15] Li J., Li D., Yang D., Huo R., Chen X., Xu Y., Dai W., Zhou D., Zhao X. (2020). Co-existing cerebrovascular atherosclerosis predicts subsequent vascular event: A multi-contrast cardiovascular magnetic resonance imaging study. J. Cardiovasc. Magn. Reson..

[16] Wabnitz A.M., Turan T.N. (2017). Symptomatic Carotid Artery Stenosis: Surgery, Stenting, or Medical Therapy?. Curr. Treat. Options Cardiovasc. Med..

[17] Campos A.M., Moura F.A., Santos S.N., Freitas W.M., Sposito A.C., Brasilia Study on Healthy Aging and Brasilia Heart Study (2017). Sarcopenia, but not excess weight or increased caloric intake, is associated with coronary subclinical atherosclerosis in the very elderly. Atherosclerosis.

[18] Lee W. (2014). General principles of carotid Doppler ultrasonography. Ultrasonography.

[19] Geroulakos G., Ramaswami G., Nicolaides A., James K., Labropoulos N., Belcaro G., Holloway M. (1993). Characterization of symptomatic and asymptomatic carotid plaques using high-resolution real-time ultrasonography. Br. J. Surg..

[20] Skilton M.R., Sérusclat A., Sethu A.H.A.U., Brun S., Bernard S., Balkau B., Moulin P., Bonnet F. (2009). Noninvasive Measurement of Carotid Extra-Media Thickness: Associations with Cardiovascular Risk Factors and Intima-Media Thickness. JACC Cardiovasc. Imaging.

[21] Costa R.M., Neves K.B., Tostes R.C., Lobato N.S. (2018). Perivascular Adipose Tissue as a Relevant Fat Depot for Cardiovascular Risk in Obesity. Front. Physiol..

[22] Carlini N.A., Harber M.P., Fleenor B.S. (2021). Age-related carotid extra-media thickening is associated with increased blood pressure and arterial stiffness. Clin. Physiol. Funct. Imaging.

[23] Hu H., Garcia-Barrio M., Jiang Z.-S., Chen Y.E., Chang L. (2021). Roles of Perivascular Adipose Tissue in Hypertension and Atherosclerosis. Antioxid. Redox Signal..

[24] Choi H.L., Au J.S., MacDonald M.J. (2017). Carotid extra-media thickness increases with age, but is not related to arterial stiffness in adults. Artery Res..

[25] Houben A.J., Eringa E.C., Jonk A.M., Serne E.H., Smulders Y.M., Stehouwer C.D. (2012). Perivascular Fat and the Microcirculation: Relevance to Insulin Resistance, Diabetes, and Cardiovascular Disease. Curr. Cardiovasc. Risk Rep..

[26] Jeong S., Yoon M. (2009). Fenofibrate inhibits adipocyte hypertrophy and insulin resistance by activating adipose PPARα in high fat diet-induced obese mice. Exp. Mol. Med..

[27] Raggi P., Gadiyaram V., Zhang C., Chen Z., Lopaschuk G., Stillman A.E. (2019). Statins Reduce Epicardial Adipose Tissue Attenuation Independent of Lipid Lowering: A Potential Pleiotropic Effect. J. Am. Heart Assoc..

[28] Hassan M., Said K., Rizk H., ElMogy F., Donya M., Houseni M., Yacoub M. (2016). Segmental peri-coronary epicardial adipose tissue volume and coronary plaque characteristics. Eur. Heart J.—Cardiovasc. Imaging.

[29] Moradi M., Talebi V. (2023). Evaluation of epicardial adipose tissue by coronary multi-detector computed tomography: An independent predictor of obstructive coronary artery disease. Egypt. J. Radiol. Nucl. Med..

[30] Baradaran H., Myneni P.K., Patel P., Askin G., Gialdini G., Al-Dasuqi K., Kamel H., Gupta A. (2018). Association Between Carotid Artery Perivascular Fat Density and Cerebrovascular Ischemic Events. J. Am. Heart Assoc..

[31] Balcer B., Dykun I., Schlosser T., Forsting M., Rassaf T., Mahabadi A.A. (2018). Pericoronary fat volume but not attenuation differentiates culprit lesions in patients with myocardial infarction. Atherosclerosis.

[32] Kuneman J.H., van Rosendael S.E., van der Bijl P., van Rosendael A.R., Kitslaar P.H., Reiber J.H.C., Jukema J.W., Leon M.B., Marsan N.A., Knuuti J. (2023). Pericoronary Adipose Tissue Attenuation in Patients with Acute Coronary Syndrome Versus Stable Coronary Artery Disease. Circ. Cardiovasc. Imaging.

[33] Farias-Itao D.S., Pasqualucci C.A., Nishizawa A., da Silva L.F.F., Campos F.M., Bittencourt M.S., da Silva K.C.S., Leite R.E.P., Grinberg L.T., Ferretti-Rebustini R.E.d.L. (2019). B Lymphocytes and Macrophages in the Perivascular Adipose Tissue Are Associated with Coronary Atherosclerosis: An Autopsy Study. J. Am. Heart Assoc..

[34] Saba L., Zucca S., Gupta A., Micheletti G., Suri J.S., Balestrieri A., Porcu M., Crivelli P., Lanzino G., Qi Y. (2020). Perivascular Fat Density and Contrast Plaque Enhancement: Does a Correlation Exist?. Am. J. Neuroradiol..

