# Supplementary material for: Peri-Carotid Adipose Tissue and Atherosclerosis at Carotid Bifurcation
Source: J Cardiovasc Dev Dis. 2024 Feb 11;11(2):58. doi: 10.3390/jcdd11020058 (PMC10889387; doi:10.3390/jcdd11020058)
Supplement: Supplementary file 1 [file jcdd-11-00058-s001.zip › jcdd-2808407-supplementary.pdf]

**SUPPLEMENTAL MATERIAL**

**Table S1** - Cardiovascular risk factors and comorbidities of the studied population

| **Cardiovascular risk factors** | **Studied Population** (n=177) |
|---|---|
| Male (n; %) | 141; 79.66 |
| Hypertension (n; %) | 121; 68.36 |
| Smoker/ex-smoker (n; %) | 112; 63.28 |
| Smoking load (PY) | 28.15 ± 31.86 |
| Dyslipidemia (n; %) | 110; 62.15 |
| Diabetes (n; %) | 66; 37.29 |
| HbA1c (%) | 6.53±3.01 |
| Coronary artery disease (n; %) | 23; 12.99 |
| **Medication** | |
| Statins (n; %) | 143; 80.79 |
| Fibrate (n; %) | 14; 7.91 |
| Ezetimibe (n; %) | 9; 5.09 |
| Antiplatelet (n; %) | 126; 71.19 |
| ACEi/ARA (n; %) | 53; 29.94 |
| Beta-blockers (n; %) | 38; 21.47 |
| Calcium channel blockers (n; %) | 51; 28.81 |

**Table S2** - Atherosclerotic characteristics at the right and left carotid bifurcation: grade of carotid stenosis, type of carotid atherosclerotic plaque and type of plaque surface

| **Carotid characteristics** | | **Studied Population** (n=177) | |
|---|---|---|---|
| | | **Right side** | **Left side** |
| IMT (mm) | | 0.90 ± 0.31 | 0.89 ± 0.26 |
| Presence of plaques (n; %) | | 122; 68.93 | 120; 67.80 |
| Number of plaques/person | | 1.46 ± 1.39 | 1.44 ± 1.31 |
| Area of the largest plaque ($mm^2$) | | 23.46 ± 31.48 | 20.86 ± 30.65 |
| Stenosis (n; %) | ≥ 50% | 21; 11.86 | 13; 7.34 |
| | ≥ 70% | 21; 11.86 | 7; 3.95 |
| Type of carotid plaque (n; %) | Type 1 | 4; 2.26 | 4; 2.26 |
| | Type 2 | 9; 7.34 | 11; 6.21 |
| | Type 3 | 59; 33.33 | 60; 33.90 |
| | Type 4 | 46; 25.99 | 45; 25.42 |
| | Type 5 | 0; 0 | 0; 0 |
| Plaque surface (n; %) | Smooth | 54; 30.51 | 53; 29.94 |
| | Irregular | 66; 37.29 | 66; 37.29 |
| | Ulcerated | 2; 1.13 | 1; 0.56 |

**Table S3** - Mean EMT and echogenicity of carotid plaques at the right and left carotid bifurcation

| Echogenicity of carotid plaques | | Present | | Non-Present | | U | p - value | r |
|---|---|---|---|---|---|---|---|---|
| | | Mean EMT (mm) | | | | | | |
| | | Median | IQR | Median | IQR | | | |
| Right side | Type 1 | 1.30 | - | 1.00 | 0.47 | 153 | .087 | .49 |
| | Type 2 | 1.13 | 0.70 | 1.00 | 0.48 | 626 | .101 | .47 |
| | Type 3 | 1.02 | 0.42 | 1.00 | 0.52 | 2752 | .705 | .11 |
| | Type 4 | 1.02 | 0.45 | 1.02 | 0.49 | 2323 | 0.614 | 0.142 |
| Left side | Type 1 | 1.35 | 0.54 | 1.00 | 0.47 | 162 | .099 | .43 |
| | Type 2 | 1.18 | 0.30 | 1.99 | 0.49 | 613 | .155 | .37 |
| | Type 3 | 0.99 | 0.59 | 1.03 | 0.43 | 2898 | .754 | .08 |
| | Type 4 | 1.04 | 0.30 | 1.01 | 0.57 | 2532 | .873 | .04 |

Note: Mean EMT was calculated as the mean between the right and the left EMT
**EMT:** extra-media thickness.

**Table S4** – Ipsilateral EMT at the right and left sides and echogenicity of the right and left carotid plaques

| Echogenicity of carotid plaques | | Present | | Non-Present | | U | p -value | r |
|---|---|---|---|---|---|---|---|---|
| | | Ipsilateral EMT (mm) | | | | | | |
| | | Median | IQR | Median | IQR | | | |
| Right side | Type 1 | 1.30 | - | 0.98 | 0.53 | 174 | .148 | .48 |
| | Type 2 | 1.01 | 0.96 | 1.00 | 0.52 | 794 | .666 | .12 |
| | Type 3 | 1.04 | 0.60 | 0.96 | 0.50 | 2555 | .518 | .19 |
| | Type 4 | 0.97 | 0.47 | 1.00 | 0.28 | 2332 | .587 | .09 |
| Left side | Type 1 | 1.42 | 0.76 | 0.95 | 0.45 | 162 | .099 | .43 |
| | Type 2 | 1.18 | 0.30 | 0.94 | 0.45 | 582 | .109 | .41 |
| | Type 3 | 0.95 | 0.50 | 0.97 | 0.47 | 2918 | .808 | .06 |
| | Type 4 | 0.96 | 0.38 | 0.95 | 0.54 | 2521 | .841 | .05 |

**EMT:** extra-media thickness.
